# Dietary niche breadth influences the effects of urbanization on the gut microbiota of sympatric rodents

**DOI:** 10.1002/ece3.9216

**Published:** 2022-09-09

**Authors:** Jason L. Anders, Alexis M. Mychajliw, Mohamed Abdallah Mohamed Moustafa, Wessam Mohamed Ahmed Mohamed, Takashi Hayakawa, Ryo Nakao, Itsuro Koizumi

**Affiliations:** ^1^ Graduate School of Environmental Science Hokkaido University Sapporo Japan; ^2^ Department of Biosciences, Center for Ecological and Evolutionary Synthesis (CEES) University of Oslo Oslo Norway; ^3^ Department of Biology Middlebury College Middlebury Vermont USA; ^4^ Department of Environmental Studies Middlebury College Middlebury Vermont USA; ^5^ Laboratory of Parasitology, Faculty of Veterinary Medicine, Graduate School of Infectious Diseases Hokkaido University Sapporo Japan; ^6^ Department of Animal Medicine South Valley University Qena Egypt; ^7^ Department of Microbiology, Biochemistry and Molecular Genetics Rutgers New Jersey Medical School Newark New Jersey USA; ^8^ Faculty of Environmental Earth Science Hokkaido University Sapporo Japan; ^9^ Japan Monkey Center Inuyama Japan

**Keywords:** artificial feeding, probiotics, rodents, sympatric species, urban ecology

## Abstract

Cities are among the most extreme forms of anthropogenic ecosystem modification, and urbanization processes exert profound effects on animal populations through multiple ecological pathways. Increased access to human‐associated food items may alter species' foraging behavior and diet, in turn modifying the normal microbial community of the gastrointestinal tract (GIT), ultimately impacting their health. It is crucial we understand the role of dietary niche breadth and the resulting shift in the gut microbiota as urban animals navigate novel dietary resources. We combined stable isotope analysis of hair and microbiome analysis of four gut regions across the GIT to investigate the effects of urbanization on the diet and gut microbiota of two sympatric species of rodents with different dietary niches: the omnivorous large Japanese field mouse (*Apodemus speciosus*) and the relatively more herbivorous gray red‐backed vole (*Myodes rufocanus*). Both species exhibited an expanded dietary niche width within the urban areas potentially attributable to novel anthropogenic foods and altered resource availability. We detected a dietary shift in which urban *A. speciosus* consumed more terrestrial animal protein and *M. rufocanus* more plant leaves and stems. Such changes in resource use may be associated with an altered gut microbial community structure. There was an increased abundance of the presumably probiotic *Lactobacillus* in the small intestine of urban *A. speciosus* and potentially pathogenic *Helicobacter* in the colon of *M. rufocanus*. Together, these results suggest that even taxonomically similar species may exhibit divergent responses to urbanization with consequences for the gut microbiota and broader ecological interactions.

## Graphical abstract

We compared the gut microbial community of sympatric rodents (*Apodemus speciosus* and *Myodes rufocanus*) between urban and natural populations. We found that a dietary shift of urban animals is partially related to host species‐specific changes in the gut microbiome.

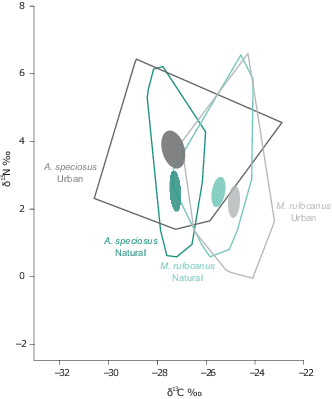

## 1 INTRODUCTION

We live in an increasingly urbanized world, and the formation of cities is accompanied by ongoing shifts in the ecology and life history of wildlife that inhabit these areas, which span some of the most biodiverse regions on the planet (Güneralp et al., 2020; Seto et al., 2012; Shochat et al., 2006). Urbanization can negatively impact the health and survival of animals (Murray et al., 2019) through habitat fragmentation (Beninde et al., 2015; Faeth et al., 2011), artificial feeding (Murray et al., 2015), and pollution (Isaksson, 2015). Importantly, the high degree of human–wildlife interactions within urban areas can increase the transmission risk of zoonotic parasites and diseases such as *Echinococcus*, hantavirus, and Lyme disease (Bradley & Altizer, 2006; Mackenstedt et al., 2014) and may be exacerbated by an associated loss of biodiversity (Destoumieux‐Garzón et al., 2018; Faeth et al., 2011). Therefore, fostering conditions that support biodiverse ecosystems is not only important from a conservation point of view, but it also extends into public health discourse (i.e., One Health; Destoumieux‐Garzón et al., 2018).

One area of recent but rapidly growing interest is how urbanization affects the gut microbiota of wildlife (Fuirst et al., 2018; Littleford‐Colquhoun et al., 2019; Murray et al., 2020; Phillips et al., 2018; Sugden et al., 2020; Teyssier et al., 2018, 2020). The gut microbial communities of animals play a pivotal role in development (Fraune & Bosch, 2010), nutritional uptake (Hooper et al., 2002), and general immune system function (Schluter et al., 2020). Therefore, any disruption to the gut microbial community (a condition known as dysbiosis) may adversely impact numerous aspects of the host's physiology and life history, ultimately affecting their health and survival (Logan et al., 2016). For example, diet simplification due to forest fragmentation can decrease gut microbial diversity (Amato et al., 2013) and negatively affect immune system function (de Paiva et al., 2016). Dietary shifts in urban animals resulting from artificial feeding, especially of low‐quality foods (Murray et al., 2015), may cause a detrimental shift in the microbial community structure within the gut that can induce numerous health issues such as obesity or inflammatory bowel disease (Chin et al., 2000; Singh et al., 2017). Furthermore, urbanization can lead to higher levels of both acute and chronic stress due to chemical, noise, and light pollution that can alter the gut microbiota and negatively impact host health (Gao et al., 2018; Isaksson, 2015).

A handful of recent studies have shown changes in the gut microbial community of urban animals as compared to conspecifics in less urbanized environments, but the degree and direction were equivocal among host taxonomic groups and study area (Fuirst et al., 2018; Littleford‐Colquhoun et al., 2019; Murray et al., 2020; Phillips et al., 2018; Sugden et al., 2020; Teyssier et al., 2018, 2020). Not only does each species of animal exhibit a unique response to urbanization due to specific life history and ecological traits in turn impacting the gut microbiota, but the ecological impacts of urbanization are not identical among cities making among study comparisons difficult. Importantly, no study has compared the gut microbial communities between sympatric species with different dietary niches within the same urban environments. Because dietary habits determine the likelihood of utilizing novel urban resources (Pagani‐Núñez et al., 2019) in turn affecting the gut microbiota and impacting host adaptability to highly modified environments, understanding the role of dietary niche breadth is crucial for predicting the effects of urbanization on the gut microbiota. We expected that dietary generalists are more likely to flexibly shift their diet in urban environments, thereby strongly altering their gut microbiota more so than species with a more restrictive diet.

We investigated the gut microbial community in four regions (small intestine, cecum, colon, and rectum) of the gastrointestinal tract (GIT) of two sympatric species of rodent in urban areas as compared to conspecifics in more natural environments (minimally modified/developed). Specifically, we were interested if a difference in dietary niche would cause a differential response to novel anthropogenic food resources that could be linked to changes in the gut microbiota. The large Japanese field mouse (*Apodemus speciosus*) and the gray red‐backed vole (*Myodes rufocanus*) belong to the same taxonomic clade Muroidea and occupy the same habitat patches throughout the island of Hokkaido, Japan (Kaneko et al., 1998; Saitoh et al., 2007). They reach a similar body size with a maximum weight of 60 g and 50 g for *A. speciosus* and *M. rufocanus*, respectively (Kaneko et al., 1998; Saitoh et al., 2007). While both species are omnivorous, *A. speciosus* preferentially consumes seeds, nuts, and insects, while *M. rufocanus* is more restricted with a diet predominantly composed of herbaceous plants (Kaneko et al., 1998; Tatsukawa & Murakami, 1976). We predicted that urban *A. speciosus* populations would have a more expanded dietary niche width than *M. rufocanus* as compared to conspecifics in a more natural habitat. This expansion should have a positive effect on gut microbiome alpha diversity in all four regions of the GIT as compared to natural conspecifics. We expected the largest impact in the lower GIT (i.e., cecum, colon, and rectum) where fermentation of plant polysaccharides occurs and the host immune system has a diminished role in shaping the microbial community (Donaldson et al., 2016). Furthermore, because novel food items are likely to require a shift in the digestive capabilities of the microbial flora, we expected urban *A. speciosus* to exhibit a larger change in the microbiota (Carmody et al., 2015; David et al., 2014). Even if the general category of dietary source (e.g., insects or plants) does not change in the urban environment, the specific plant and insect species consumed may (Beninde et al., 2015; Faeth et al., 2011). This study aimed to generate generalized insights into the conditions that lead to dietary changes within urban environments and the subsequent response of the gut microbiota, ultimately impacting the adaptability of animals to highly modified environments.

## 2 METHODS

### 2.1 Fieldwork and gut content sampling

In October 2019, two sympatric species of rodent, the large Japanese field mouse (*A. speciosus*, *n* = 83) and the gray red‐backed vole (*M. rufocanus*, *n* = 93), were captured in forest fragments within two urban parks (i.e., Kaguraoka koen and Shunkodai koen) in the city of Asahikawa and one park (i.e., Maruyama koen) in the city of Biei, as well as four natural sites (i.e., Shirakkeyama, Chitoseyama, Harushinai, and Mukoyama) within the surrounding Kamikawa Chubu National forest in central Hokkaido, Japan (Figure S1 and Table S1). The urban parks are surrounded by residential areas with Kaguraoka koen notably situated next to the central built‐up area of Asahikawa city. All parks are actively managed and heavily used by the public. Our natural sites were located in the middle of the forest at least 1 km from any agricultural or built‐up areas preventing exposure to human impacts as the species home ranges are much smaller (Ims, 1987; Oka, 1992). All natural sites were higher in elevation than any potential pollution runoff but below 500 m in order to avoid altitudinal variation in the gut microbiome (Suzuki et al., 2019). Both the national forest and managed forest fragments within the urban parks were primarily composed of deciduous trees such as birch, oak, and walnut while the underbrush was mostly dwarf bamboo (*Sasa kurilensis*) with some various small leafy plants. At each site, we deployed two or three trap grids of Sherman traps (H.B. Sherman Traps, Inc.) baited with oatmeal and placed in a 4 × 10 grid pattern with each trap 10 m apart. We checked all traps within 1 h after sunrise for two to three consecutive days, and replaced any trap containing an animal with a fresh one.

Animals were transported to the Department of Parasitology at Asahikawa Medical University in Asahikawa where they were euthanized, identified, weighed (g), and sexed by the presence of ovarian tubes for females and testes for males. We classified each individual as adult or subadult according to average body weight at maturation for each species (Kuwahata, 1984; Oh & Mori, 1998), and body condition was estimated by dividing the log of body weight by the log of body length (Labocha et al., 2014). After removal of the GIT immediately after euthanization, gut content was collected from the ileum in the small intestine, the cecum, and the descending colon, as well as fecal matter from the rectum using a small steel spatula and utilizing sterilization‐based laboratory techniques. Samples were placed in a −80°C freezer within 1 h of collection where they were stored until transfer to the Laboratory of Parasitology in the Faculty of Veterinary Medicine at Hokkaido University, Sapporo, Japan for DNA processing. Finally, hair was collected from the outer hind legs and dried under a fume hood for 48 h for use in stable isotope analysis. Experimental design and handling of animals was approved by the Institutional Animal Care and Use Committee of the national University Corporation Hokkaido University (reference number 15‐0121) and carried out in accordance with their guidelines.

### 2.2 Stable isotope analysis

We utilized stable isotopes of carbon (δ^13^C) and nitrogen (δ^15^N) to reconstruct the diet of our target host species, a widely applied technique in ecological studies (Baltensperger et al., 2015; Ben‐David & Flaherty, 2012). Here, we analyzed stable isotope values of hair as a proxy for long‐term resource use to determine the effect of dietary habits on the gut microbial community. Because all individuals had undergone their winter molt, stable isotope values should represent their diet from September to early October just prior to capture. Before analysis, hair was washed using a chloroform: methanol solution (2:1 v/v) for removal of surface oils, then rinsed in distilled water and dried in an oven at 60°C for 48 h. We wrapped 0.5 mg of hair from each individual in tin capsules and analyzed stable isotope ratios using an elemental analyzer (Flash EA 1112, Thermo Fisher) coupled to an isotopic ratio mass spectrometer (IRMS, Delta V Plus, Thermo Fisher). Standardization of isotopic ratios is based on Vienna Pee Dee Belemnite (VPDB) for δ^13^C and atmospheric nitrogen (AIR) for δ^15^N and presented as parts per mil (‰).

### 2.3 Extraction, PCR, and high‐throughput sequencing

Following Hayakawa et al. (2018), DNA was extracted from gut content and fecal matter using the QIAamp fast DNA stool mini kit (Qiagen) after bead beating for 3 min with 1 mg of 0.1 mm and four 3 mm silica/zirconia beads. PCR amplification of the V3–V4 region of the 16S rRNA gene was performed using the 314F/805R universal primers (Klindworth et al., 2013). DNA extraction and PCR amplification were performed under sterile conditions and a negative control was included for each step in each batch of samples. Finally, high‐throughput sequencing was done on a MiSeq (Illumina, San Diego) 300 bp paired‐end platform using a Reagent kit v3 after library preparation using Nextera XT Index Kit v2 set A, B, C, or D following the manufacturer's instructions. A more detailed description of the DNA processing methods can be found in Anders et al. (2021), and raw sequences have been submitted to the DNA database of Japan (DDBJ) with the accession numbers DRA011343 and DRA011772.

### 2.4 Data analysis

We used a pairwise Wilcoxon rank‐sum test in the statistical program R (version 4.0.2; R Core Team, 2020) to compare δ^13^C and δ^15^N values between species and habitat conditions as the data deviated from normality. We then investigated the δ^13^C and δ^15^N isotopic niche space on an xy‐plane occupied by urban and natural populations by calculating standard ellipse area (small sample size corrected; SEA_c_) for each host species in the R package SIBER (Jackson et al., 2011). A Bayesian multivariate distribution was fit to each host species in urban and natural habitats using Gibbs sampling technique over 20,000 iterations with a burn in of 1000 implemented in the R package rjags (Plummer, 2019). We compared the niche size of urban and natural populations using maximum likelihood estimates of SEA_c_ and by calculating the posterior distribution of the covariance matrix generating Bayesian SEA (SEA_b_). For pairwise comparisons between the ellipse sizes of the different species and ecosystems, the probability that a given ellipse had a larger posterior distribution was calculated using paired posterior draws where the proportion of draws that were larger serves as a proxy for probability. We also calculated the proportional overlap of maximum likelihood fitted standard ellipses to quantify the degree of niche space overlap between the two species or habitat type, where 0 indicates no overlap and 1 complete overlap.

We quantified the differential contributions of food resource categories to both species in each habitat type using a stable isotope mixing model in the R package SIMMR (Parnell, 2020). Because no specific trophic enrichment factors are available for these rodent species, we used an average value of rodent hair isotopic offsets (δ^15^N 2.7 ± 1.67‰ and δ^13^C 2.4 ± 1.01‰) as utilized in a study of similar focal taxa (i.e., rodents; Baltensperger et al., 2015). Isotopic values of potential food items were taken from previously published studies in Hokkaido including various C3 plant leaves and stems (δ^13^C −31.65 ± 0.62‰ and δ^15^N −1.98 ± 1.32‰; Osaki et al., 2019), C3 fruits and nuts including acorns (δ^13^C −28.15 ± 1.12‰ and δ^15^N −2.13 ± 0.33‰; Osaki et al., 2019), C4 corn (δ^13^C −10.19 ± 0.04‰ and δ^15^N −2.13 ± 0.33‰; Matsubayashi et al., 2014), and terrestrial animals including herbivorous mammals and insects (δ^13^C −26.3 ± 0.5‰ and δ^15^N 3.7 ± 1.5‰; Matsubayashi et al., 2014). Where necessary, δ^13^C values were corrected for the Suess effect of atmospheric carbon depletion using a year‐specific correction to 2019 values (Long et al., 2005). Model fit was evaluated through assessment of Gelman diagnostics of MCMC convergence and a posterior predictive check.

Paired‐end sequence reads of the microbiome from gut content and fecal matter were demultiplexed then trimmed to remove the primers and low‐quality regions, quality filtered, and merged, using the DADA2 pipeline in QIIME2 version 2020.2 (Bolyen et al., 2019; Callahan et al., 2016), producing a table of amplicon sequence variants (ASVs). Few sequence reads were found within our negative controls (Average = 130 ± 168.98 SD), thereby limiting their use for decontaminating our samples. Therefore, potential contaminant sequence reads were identified using the frequency method with a threshold of 0.1 in the decontam package in R (Davis et al., 2018). A total of 195 potential contaminant sequence reads were identified and the frequency plots of each were checked for confirmation. We then manually confirmed the presence of each potential contaminant within our negative controls. Four sequence reads were found to be highly prevalent and highly abundant within our samples yet rare or nonexistent within our negative controls. Therefore, 191 potential contaminant sequences were removed using sequence identifiers in QIIME2. The SILVA classifier (release 138) was used for taxonomic classification of each ASV using the feature‐classifier plugin (Bokulich et al., 2018). Only bacterial ASVs identified at phylum level or below were kept for further analyses; all others were removed. Per sample raw read counts and relative abundances of each bacterial taxa can be found in Appendix S1. A rooted phylogenetic tree was generated using the FastTree method with the MAFFT plugin in QIIME2 (Price et al., 2010).

Based on alpha rarefaction analysis, samples were rarified to a sampling depth of 10,000 reads to calculate diversity metrics to maximize coverage of the variation in the microbiome while limiting sample omission, similar to Anders et al. (2021). A total of six samples (three small intestine and one feces from *M. rufocanus* and two small intestine from *A. speciosus*) with low sequence counts were excluded from diversity analyses. Alpha diversity was quantified using the number of ASVs and Faith's phylogenetic diversity (PD). To analyze beta‐diversity comparing habitat type, unweighted and weighted UniFrac dissimilarity matrices were generated in Qiime2.

We investigated whether alpha diversity of the gut microbiome of rodents is altered within the urban environment by developing a linear mixed‐effects model (LME) in which the response variable was log‐transformed alpha diversity, the random effect was site, and the explanatory variables were habitat type (i.e., urban or natural), sex, age (adult or subadult), δ^13^C, and δ^15^N using the nlme package in R (Pinheiro et al., 2020). This was repeated for each gut region in each host species. Body condition was not included in the models because we found no difference between urban and natural populations in neither *A. speciosus* (ANOVA, *F* = 0.186, *p* = .667) nor *M. rufocanus* (ANOVA, *F* = 0.333, *p* = .565). Beta‐diversity was first visualized using principle coordinate analysis (PCoA) plots for each gut region in each species performed in the R package phyloseq (McMurdie & Holmes, 2013). PERMANOVA analysis was then conducted to determine the impact of habitat type, sex, age, δ^13^C, and δ^15^N on beta‐diversity within each gut region of both rodent species using the adonis2 function with the margin=“by” option to determine the marginal effect of each in the vegan package in R (Oksanen et al., 2020). Within habitat‐type variation of the gut microbial community within each gut region was analyzed using PERMDISP with 999 permutations in the vegan package in R for both unweighted and weighted UniFrac dissimilarity matrices (Oksanen et al., 2020).

Linear discriminant analysis effect size (LEfSe) was used to compare relative abundances of the different microbial genera between urban and natural populations of both *A. speciosus* and *M. rufocanus*. This was done for each gut region separately within each species using the Huttenhower lab Galaxy pipeline where class was habitat type (Segata et al., 2011). We also applied ANCOM‐II for differential abundance analyses, the results of which are reported within Appendix S2.

## 3 RESULTS

### 3.1 Host capture and gut content

We captured 42 and 41 *A. speciosus*, and 43 and 50 *M. rufocanus* from natural and urban habitats, respectively (Table S1). A total of 245 gut content and fecal matter samples were collected from *A. speciosus* and 238 from *M. rufocanus* for microbiome analysis (Table S2). After quality filtering, 21,820,759 high quality reads were obtained with 11,264,730 (average of 45,978 ± 12,453 SD per sample) from *A. speciosus* and 10,556,029 (average of 44,167 ± 16,512 SD per sample) from *M. rufocanus*.

### 3.2 Expanded dietary niche width and dietary shift

**FIGURE 1:**
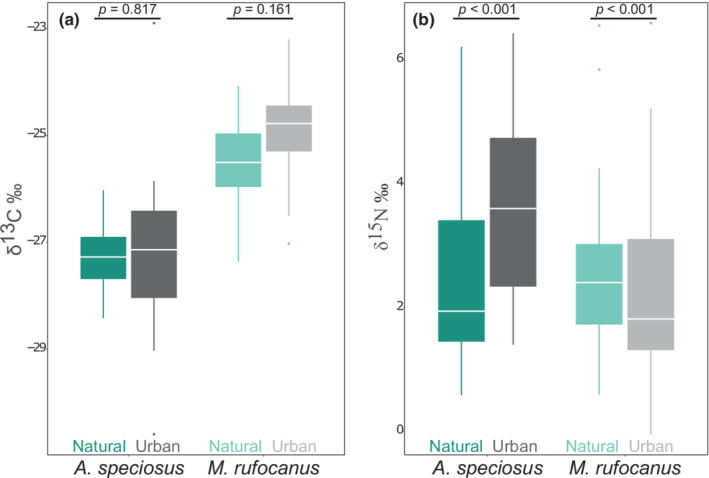
Comparison of (a) δ^13^C and (b) δ^15^N between species and populations in urban and natural areas. The *p*‐values are based on pairwise Wilcoxon rank‐sum test.

We found significant differences in δ^15^N (*p* < .001) between natural and urban populations of both species, but not δ^13^C (*p* > .05; Figure 1). There was a significant difference in both δ^13^C and δ^15^N values between species in the urban areas (*p* < .001), but only δ^13^C was significantly different within the natural sites (Figure 1; Table S3).

**FIGURE 2:**
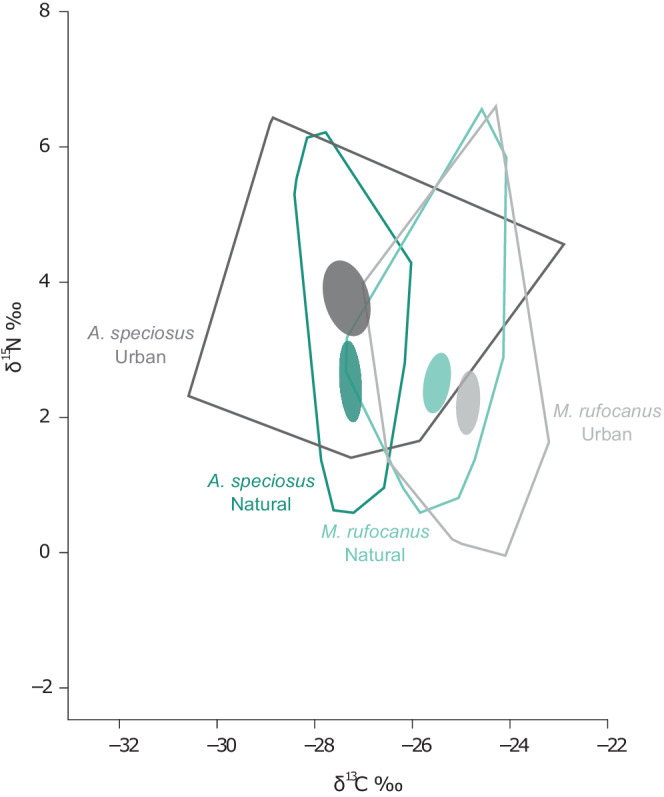
Total area convex hulls (solid lines) and 95% confidence intervals around bivariate means (filled ovals) for natural (green) and urban (gray) populations of *Apodemus speciosus* and *Myodes rufocanus* as calculated in SIBER.

Isotopic niche width (standard ellipse area, sample size corrected; SEA_c_) found that urban *A. speciosus* (SEA_c_ = 5.78) had an isotopic niche almost twice as large as those in the natural habitat (SEA_c_ = 2.9) with a .9988 probability of the urban ellipse being larger than the natural based on paired posterior draws of the Bayesian distribution (Figure 2; Figure S2 and Table S4). *M. rufocanus* also exhibited a larger niche width in the urban parks although to a lesser degree than *A. speciosus* supporting our first hypothesis, with an SEA_c_ of 2.68 and 3.01 in the natural and urban areas respectively, and a probability of .7138 that the urban ellipse was larger (Figure 2; Figure S2). Furthermore, the degree of pairwise overlap between natural and urban populations was larger for *M. rufocanus* (.59) than *A. speciosus* (.39). *A. speciosus* also had larger SEA_b_ values than *M. rufocanus* in both the urban (.9993 probability) and natural (.6645 probability) habitats.

Estimating the proportion of each food item in the diet of both species of rodent found that terrestrial animal protein made up a marginally larger portion of the diet of *M. rufocanus* (32.2%) than *A. speciosus* (28.7%) within the natural habitat, though this slim margin may be due to the choice of input food sources available from the literature (Table 1). *A. speciosus* shifted toward consuming more terrestrial animal protein within the urban parks (43.2% of their diet) as compared to their natural conspecifics (28.7%) with a slight shift away from C3 plant leaves and stems (60.4% to 52.4% in natural and urban, respectively; Table 1). Urban *M. rufocanus* exhibited the opposite trend with a shift toward C3 plants (35.9% to 60.6% in natural and urban respectively) while consumption of terrestrial animal protein slightly decreased from 32.2% to 28.26% (Table 1). Both species were consuming less C3 fruits and nuts in the urban parks as compared to their natural conspecifics (Table 1).

**TABLE 1:** The average proportion of each food item in the diet of *Apodemus speciosus* and *Myodes rufocanus* in natural and urban populations (percent ± standard deviation).

	*A. speciosus*		*M. rufocanus*	
	Natural	Urban	Natural	Urban
C3 Plants	60.4 ± 3.9	52.4 ± 4.2	35.9 ± 9.8	60.6 ± 3.9
C3 Fruits & nuts	9.1 ± 4.8	3.4 ± 2.4	25.5 ± 11.6	8.9 ± 4.8
C4 Corn	1.8 ± 0.8	1.0 ± 0.6	6.5 ± 2.2	1.9 ± 0.9
Terrestrial animals	28.7 ± 3.7	43.2 ± 3.9	32.2 ± 3.4	28.6 ± 3.7

### 3.3 No change in alpha diversity in urban populations

**FIGURE 3:**
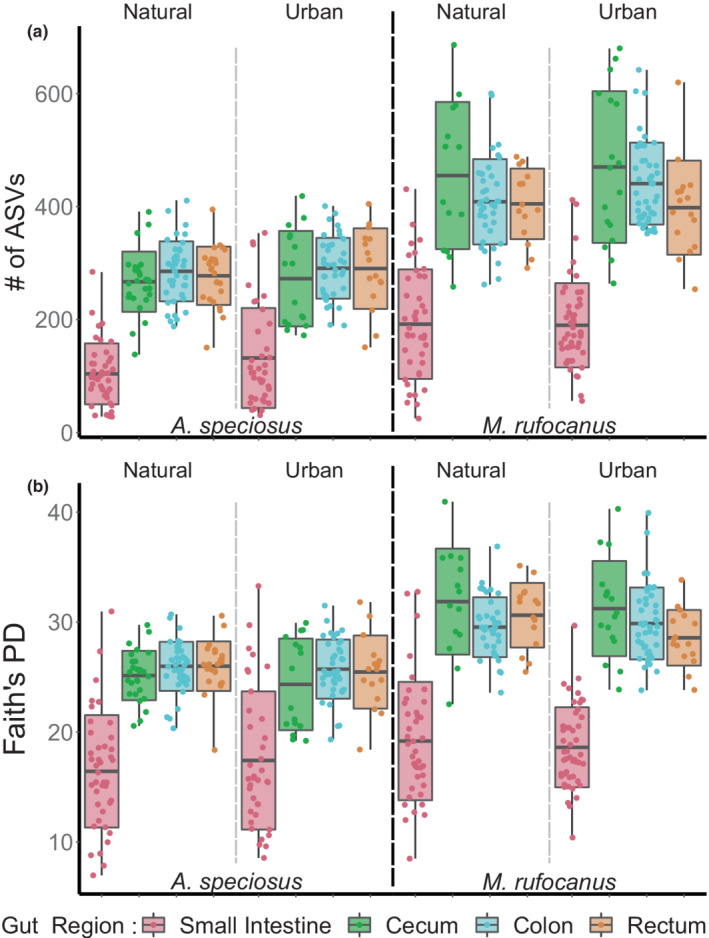
Alpha diversity along the gastrointestinal tract of *Apodemus speciosus* and *Myodes rufocanus* in natural and urban populations according to (a) the number of ASVs and (b) Faith's PD. The black dashed line separates host species and the gray dashed line separates habitat type.

Inconsistent with our second prediction, we found that habitat type had no effect on alpha diversity for any diversity index in any gut region for either host species (all *p* > .05, Figure 3; Tables S5 and S6). Interestingly, neither δ^13^C nor δ^15^N significantly impacted gut microbiome alpha diversity in any gut region of the omnivorous *A. speciosus*, despite there being significantly higher δ^15^N values reflected in the hair of urban individuals (Tables S5 and S6). In the more herbivorous *M. rufocanus*, δ^13^C had a significantly negative relationship with Faith's PD and the number of ASVs in the cecum and rectum (LME: all *p* < .05; Tables S5 and S6), as well as in the colon for Faith's PD (LME: *b* = −0.033 ± 0.015, *p* = .035, Table S6), and in the small intestine for the number of ASVs (LME: *b* = −0.22 ± 0.077, *p* = .006; Table S5). On the other hand, δ^15^N had a significantly positive effect on Faith's PD and the number of ASVs in both the colon and rectum (all *p* < .05, Tables S5 and S6). Males had significantly higher microbial alpha diversity in the cecum, colon, and rectum of *A. speciosus*, but not in the small intestine; nor was there an effect of sex in any gut region in *M. rufocanus* (Tables S5 and S6). Age, on the other hand, significantly affected alpha diversity in *M. rufocanus*, particularly in the colon and small intestine, but had no effect in *A. speciosus* (Tables S5 and S6).

### 3.4 Habitat type impacts gut microbiome beta‐diversity

**FIGURE 4:**
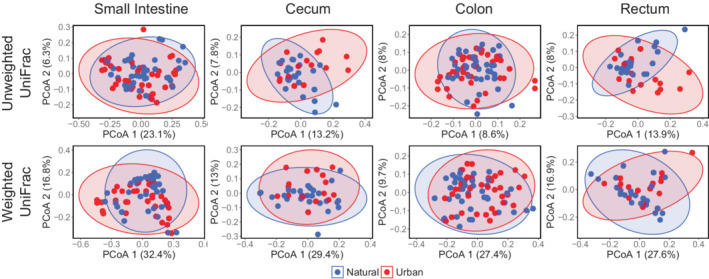
PCoA plots of the gut microbial community along the gastrointestinal tract of *Apodemus speciosus* based on unweighted and weighted UniFrac dissimilarity metrics. Blue are individuals from the natural areas and red are urban.

**FIGURE 5:**
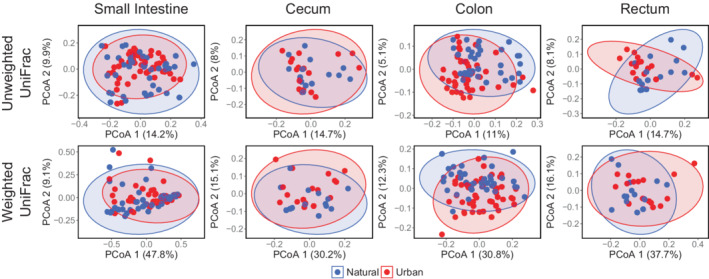
PCoA plots of the gut microbial community along the gastrointestinal tract of *Myodes rufocanus* based on unweighted and weighted UniFrac dissimilarity metrics. Blue are individuals from the natural areas and red are urban.

Visualization of the gut microbial community composition using PCoA plots exhibited a high degree of overlap in the clustering of urban and natural populations in all gut regions in both host species, though not entirely (Figures 4 and 5). Interestingly, the cluster area size of urban individuals was the same size or slightly larger than the cluster of individuals from the natural habitats for the cecum, colon, and rectum of both species for both unweighted and weighted UniFrac (Figures 4 and 5). This was also the case in the small intestine of *A. speciosus* (Figure 4), but not *M. rufocanus* where the cluster area of individuals from the urban parks was smaller than those from the national forest (Figure 5). This trend was largely unconfirmed by PERMDISP as a significant difference in dispersion was only found in the small intestine of *M*. rufocanus for unweighted UniFrac (*F* = 4.027, *p* = .039, Table S7). There was also an inverted relationship between PCoA 1 and PCoA 2 for unweighted UniFrac in the cecum and rectum of urban *A. speciosus* as compared to their natural conspecifics, but only in the rectum according to weighted UniFrac (Figure 4).

Using PERMANOVA to test for the effect of habitat type on gut microbiota beta‐diversity, we found a significant effect of habitat type in all four gut regions of both *A. speciosus* and *M. rufocanus* for unweighted UniFrac (all *p* < .05, Tables S8 and S9). There was also a significant effect in the colon (PERMANOVA: *R* ^2^ = .038, *F* = 3.173, *p* = .004) and small intestine (PERMANOVA: *R* ^2^ = .0299, *F* = 2.303, *p* = .04) but not the cecum (PERMANOVA: *R* ^2^ = .024, *F* = 1.076, *p* = .36) or rectum (PERMANOVA: *R* ^2^ = .042, *F* = 1.768, *p* = .078) in *A. speciosus* based on weighted UniFrac (Table S8). In *M. rufocanus* there was a significant effect of habitat type in the small intestine (PERMANOVA: *R* ^2^ = .051, *F* = 4.519, *p* = .009), cecum (PERMANOVA: *R* ^2^ = .068, *F* = 2.066, *p* = .039) and colon (PERMANOVA: *R* ^2^ = .034, *F* = 2.799, *p* = .014), but not the rectum (PERMANOVA: *R* ^2^ = .068, *F* = 2.054, *p* = .064) according to weighted UniFrac (Table S9). Similar to alpha diversity, δ^13^C and δ^15^N values had a greater effect on gut microbiome beta‐diversity in *M. rufocanus* than *A. speciosus*. Specifically, δ^13^C significantly impacted beta‐diversity in all four gut regions based on unweighted UniFrac with the *R* ^2^ value twice as large in the cecum and rectum than in the small intestine and colon (PERMANOVA: all *p* < .05). Only in the small intestine was there a significant effect of δ^13^C according to weighted UniFrac (PERMANOVA: *R* ^2^ = .033, *F* = 2.919, *p* = .03; Table S9). δ^15^N also significantly impacted beta‐diversity in the cecum, colon, and rectum of *M. rufocanus* based on unweighted UniFrac (all *p* < .05; Table S9), but no effect in any gut region was found for weighted UniFrac (all *p* > .05; Table S9). In *A. speciosus*, only in the colon was there a significant effect of δ^13^C on gut microbiome beta‐diversity according to unweighted UniFrac alone (PERMANVOA: *R* ^2^ = .022, *F* = 1.769, *p* = .005) while δ^15^N had no effect in any gut region (all *p* > .05, Table S8). Overall, these results support our third hypothesis predicting a change in diet associated with urbanization having a greater impact on the gut microbiota composition in the lower GIT than in the small intestine.

Unlike alpha diversity, sex had a minimal impact on beta‐diversity in either host species with it only being significant in the colon of *A. speciosus* as well as the rectum and colon of *M. rufocanus* (all *p* < .05; Tables S8 and S9). Age significantly impacted beta‐diversity in the colon of *A. speciosus* for both unweighted (PERMANVOA: *R* ^2^ = .024, *F* = 1.933, *p* = .001) and weighted UniFrac (PERMANVOA: *R* ^2^ = .049, *F* = 4.078, *p* = .001; Table S8), while it was significant in the cecum, colon, and small intestine of *M. rufocanus* based on unweighted UniFrac (all *p* < .05; Table S9).

### 3.5 Differential relative abundance of bacterial genera

**FIGURE 6:**
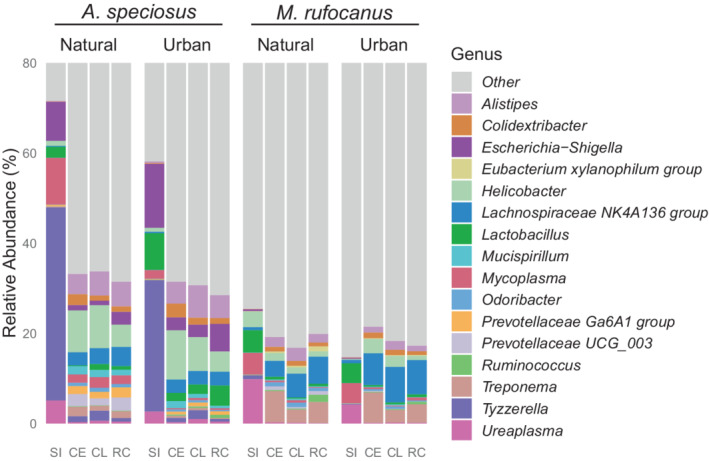
Relative abundance of microbial genera exhibiting significantly different relative abundance between habitat type based on LEfSe analysis and comprised at least 1% of the gut microbiota in at least one gut region of either host species. The category “other” indicates relative abundances of all other microbial genera regardless of statistical significance. RC, rectum; CE, cecum; CL, colon; SI, small intestine.

LEfSe analysis found multiple microbial genera in significantly higher abundance in the urban habitat as compared to the national forest or vice versa, with several particularly noteworthy genera (Tables S10 and S11). The probiotic group *Lactobacillus* in the small intestine, *Butyricicoccus* in the cecum and colon, and *Bifidobacterium* in both the small intestine and colon had significantly higher relative abundance in urban *A. speciosus* as compared to conspecifics in the natural habitats (Figure 6; Table S10). There was significantly higher abundance of the potentially pathogenic *Helicobacter* in the colon of urban *M. rufocanus*, while natural populations had higher abundance of *Helicobacter* the small intestine (Figure 6; Table S11). Although LEfSe analysis specifically tests for higher abundance of microbes, the opposite can be inferred. For example, the significantly higher relative abundance of *Alistipes* in both the cecum and colon of *M. rufocanus* and *Tyzzerella* in the small intestine of *A. speciosus* from the natural habitat means there is lower abundance in the urban parks (Figure 6; Tables S10 and S11).

## 4 DISCUSSION

To our knowledge, no previous study has contrasted the response of the gut microbiota of multiple animal species within the same urban areas (Fuirst et al., 2018; Littleford‐Colquhoun et al., 2019; Murray et al., 2020; Phillips et al., 2018; Sugden et al., 2020; Teyssier et al., 2018, 2020). Not only does each animal species harbor their own unique gut microbiota (Kohl et al., 2018), interspecific differences in ecological traits effects their interaction with novel anthropogenic environments and their associated stressors, in turn affecting the gut microbiota. Therefore, such changes are likely to be host species‐specific. Our design incorporating two sympatric rodent species with differing life histories and ecological traits in the same urban areas permits us to detect differential responses of the gut microbiota when exposed to the same environmental pressures of urbanization. We found that both species exhibited distinct changes in dietary niche possibly in response to anthropogenic food resources, and may partially explain species‐specific changes in their gut microbial communities.

### 4.1 Increased dietary niche width in urban populations

Based on stable isotopes of carbon (δ^13^C) and nitrogen (δ^15^N), both species of rodents appear to occupy an expanded dietary niche in urban parks compared with natural areas, and as expected, this pattern was more pronounced in the omnivorous *A. speciosus* (Figure 2; Figure S2). The lower degree of overlap in the isotopic niche space between natural and urban populations of *A. speciosus* suggests they may be more likely to utilize novel food resources and diverge in dietary habits than the more herbivorous *M. rufocanus*. Results similar to what we found in *A. speciosus* have been reported in several omnivorous species occupying urban habitats (Littleford‐Colquhoun et al., 2019; Murray et al., 2015; Pagani‐Núñez et al., 2019).

The increased proportion of animal protein consumed by urban *A. speciosus* as compared to their conspecifics in the national forest possibly comes from the consumption of human provided animal products. This is because urban sites in this study are heavily used parks by local citizens with barbequing and picnics particularly popular activities during late spring to autumn. Scraps of meat and other food trash were commonly seen throughout the forest fragments during field surveys and may be opportunistically consumed by *A. speciosus* as an easy energy source (Larson et al., 2020; Pagani‐Núñez et al., 2019). However, *A. speciosus* could simply be consuming more insects as artificial lighting such as street and park lamps increase insect susceptibility to predation by birds and rodents (Owens et al., 2020; Yoon et al., 2010). The difference in rodent community structure between the two habitat types may also drive the increased consumption of insects. Specifically, within the national forest, the population size of *A. speciosus* and *M. rufocanus* was inversely related at each site, but in the urban parks, they were more equal in number (Table S1) possibly due to forest fragmentation limiting dispersal even when population density is high (Sato et al., 2014). Therefore, increased interspecific competition for food resources possibly push each species to preferentially consume food items for which they are more specialized (i.e., insects for *A. speciosus* and herbaceous plants for *M. rufocanus*). This is in contrast to more free roaming animals such as coyotes and birds that have access to a much wider array of microhabitats within cities and can more readily take advantage of anthropogenic resources, thereby avoiding interspecific competition (Larson et al., 2020; Pagani‐Núñez et al., 2019; Phillips et al., 2018).

It is possible that chemicals containing high levels of δ^15^N such as some fertilizers (Bateman & Kelly, 2007) used for park management purposes are artificially elevating the isotopic values of animal hair in our study. Such chemicals could be contaminating food items or elevating δ^15^N values in plant tissue consumed by *A. speciosus* through uptake from the soil. While uncertainty remains because we did not measure the isotopic values of potential food items, such a scenario is unlikely to explain the observed dietary shift entirely because we would expect to see a similar trend in *M. rufocanus* (Figure 1).

### 4.2 No change in gut microbial alpha diversity in urban populations

Altered diets in urban areas can lead to changes in alpha diversity of the gut microbiome potentially decreasing alpha diversity in response to a simplified or low‐quality diet (Fuirst et al., 2018; Teyssier et al., 2020), or increasing diversity due to access to more diverse or novel resources (Littleford‐Colquhoun et al., 2019; Phillips et al., 2018). Lower alpha diversity of the gut microbial community is typically associated with dysbiosis (Logan et al., 2016), highlighting the need to understand how urbanization affects it. We did not detect a change in gut microbial alpha diversity in any gut region within the urban populations of either rodent species as compared to those in the natural habitat despite the wider dietary niche (Figure 3; Tables S5 and S6). It is possible that other factors not tested for such as pollution or stress have a large enough negative impact on alpha diversity in these animals to mask any positive effect of diet (Gao et al., 2018; Han et al., 2014; Isaksson, 2015). On the other hand, because our dietary niche width analysis using stable isotopes was at the population level, a more plausible explanation is that intraindividual dietary diversity remains consistent regardless of habitat type, but that interindividual variation is much greater in the urban parks due to access to novel human associated food items.

### 4.3 Microbial community composition is affected by both diet and habitat type

The significant effect of habitat type on beta‐diversity throughout the GIT of both rodent species indicates that a unique microbial community of the gut may be associated with urban populations as compared to natural conspecifics. Diet is one of the major factors affecting gut microbial community structure (David et al., 2014). Therefore, the dietary shift of urban populations in this study is a likely factor affecting beta‐diversity and overall microbial community structure as δ^13^C and δ^15^N values had an impact throughout the GIT of *M. rufocanus*, particularly within the lower GIT, as well as in the colon of *A. speciosus* (Tables S8 and S9). For example, *Bifidobacterium* was only found in the colon and small intestine of urban *A. speciosus*. This genus was nonexistent in individuals from the national forest and may be a human‐associated microbe (Table S10) because it is found in fermented foods such as natto, miso, and yogurt (Fujisawa et al., 2006) that could be consumed by *A. speciosus*. However, its exceedingly low relative abundance (average <0.02%) may indicate it is not a gut resident but was within recently consumed food. In *M. rufocanus*, we found lower abundance of *Alistipes* in the cecum of urban individuals (Figure 6; Table S11). This genus is associated with the consumption of animal protein (David et al., 2014), and their lower abundance fits with the shift in diet away from terrestrial animals, although the shift is small and an opposite trend was not was observed in *A. speciosus* (Figure 6; Tables S9 and S10).

Although stable isotope values explained variation in beta‐diversity only within the colon of *A. speciosus*, this does not exclude the effect of diet as the proportion of fats, proteins, and carbohydrates can have a profound effect on microbial community structure (David et al., 2014; Singh et al., 2017). We were unable to identify specific food items, but the higher relative abundance of *Lactobacillus* in the small intestine of urban *A. speciosus* may be explained by the elevated consumption of anthropogenic food containing animal protein (Figure 6; Table S10). Increased protein consumption has been shown to positively affect *Lactobacillus* abundance (Zhu et al., 2016). Alternatively, higher *Lactobacillus* abundance may be associated with specific food items (Sasaki et al., 2005) that are more abundant or preferentially consumed within the urban parks.

Increased abundance of *Lactobacillus* within urban populations as compared to those outside of city limits has been reported in house sparrows in Europe (Teyssier et al., 2018, 2020) and water dragons in Australia (Littleford‐Colquhoun et al., 2019). Why we did not see a similar increase in *M. rufocanus* is an important question that must be investigated further. The bacterial genus *Lactobacillus* contains many probiotic species that provide a wide range of benefits to their host including reduction of intestinal inflammation (Liu et al., 2010) and regulation of immune system function (Schluter et al., 2020). This important genus may aid in the successful adaption of animals to the urban environment by helping protect them from the adverse effects of novel stressors, thereby allowing the animals to remain healthy.

Interest has been growing in the microbial genus *Tyzzerella* because higher relative abundance has been associated with a low‐quality diet (Liu et al., 2019) and a higher lifetime risk of cardiovascular disease, obesity, and nonalcoholic fatty liver disease among other ailments (Daniel et al., 2021; Kelly et al., 2016; Wang et al., 2022). We found a notably lower relative abundance of *Tyzzerella* within the small intestine of urban *A. speciosus* (Figure 6; Table S10). It is possible that access to higher quality food items within the urban parks is causing the lower relative abundance of this genus and better overall health of the animals. But causality of disease has yet to be shown for *Tyzzerella* and its remarkably high relative abundance in both habitats indicates it may be a normal member of the gut microbiota of this rodent species.

Curiously, there was lower abundance of *Helicobacter* in the small intestine, but higher abundance in the colon of *M. rufocanus* in the urban parks as compared to those in the national forest (Figure 6). Most species of *Helicobacter* thrive within the low pH environment of the stomach and small intestine, and many species are pathogenic in both humans and animals (On et al., 2015). Those species that have been isolated from the lower GIT such as *H. hepaticus* are known to induce inflammatory bowel disease (IBD) in immunocompromised animals and are associated with a markedly different microbial community of the cecum and colon (Yang et al., 2013). Although we did not test immune system function directly, there was less interindividual variation in the gut microbiome of the small intestine within the urban populations due to species membership (Figure 5; Table S7). The small intestine is the most immunologically active location in the entire body and the host immune system plays a pivotal role in shaping the gut microbial community (Bevins & Salzman, 2011). Therefore, a similar and strong immunological shift in response to the urban environment due to elevated stress or pollution (Gao et al., 2018; Isaksson, 2015) could cause a convergence in the gut microbial community structure and leave them more susceptible to the proliferation of pathogens in the lower GIT such as *Helicobacter*. However, a lower body condition would be expected in diseased animals, yet we found no difference between urban and natural populations. *Helicobacter* may be a normal member of the gut microbial community of both rodent species as high abundance was also found throughout the lower GIT of *A. speciosus* in both habitat types (Figure 6). Such findings mirror what has previously been reported in other wild rodents without the onset of disease as opposed to laboratory animals who maintain relatively low abundance of *Helicobacter* yet are more susceptible to a variety of diseases (Bowerman et al., 2021; Rosshart et al., 2017). Future studies should investigate species‐specific immune response to urbanization and how this may affect their gut microbiome and their susceptibility to pathogens.

### 4.4 Host species‐specific response to urbanization

In comparing populations from urban and natural areas of northern Japan, we report host species‐specific and gut region‐specific changes in the gut microbial communities of two sympatric species of rodent occupying the same habitats. Some of the changes may be associated with a dietary shift that is consistent with the two species' ecological traits (i.e., omnivorous or herbivorous) and may be influenced by the consumption of novel anthropogenic food resources or an altered rodent community structure increasing interspecific competition. While we did not find a clear indication of dysbiosis in either species, there was a homogenization of the gut microbiome in the small intestine of *M. rufocanus* and higher relative abundance of a potentially pathogenic *Helicobacter* species in the lower GIT. On the other hand, urban *A. speciosus* are harboring a lower abundance of *Tyzzerella* and a higher abundance of multiple probiotic genera that may protect them from the negative effects of ecosystem modification. Investigating changes in the gut microbiota in multiple host species and gut regions within the same urban areas provides deeper insights into potential mechanisms behind such alterations that are associated with differing life histories.

## AUTHOR CONTRIBUTIONS

**Jason L. Anders:** Conceptualization (lead); formal analysis (lead); funding acquisition (lead); investigation (lead); methodology (lead); visualization (lead); writing – original draft (lead). **Alexis M. Mychajliw:** Formal analysis (equal); methodology (equal); visualization (equal); writing – review and editing (supporting). **Mohamed Abdallah Mohamed Moustafa:** Formal analysis (supporting); methodology (supporting); writing – review and editing (supporting). **Wessam Mohamed Ahmed Mohamed:** Formal analysis (supporting); visualization (supporting); writing – review and editing (supporting). **Takashi Hayakawa:** Formal analysis (supporting); methodology (supporting); resources (supporting); writing – review and editing (supporting). **Ryo Nakao:** Funding acquisition (equal); methodology (equal); resources (equal); supervision (supporting); writing – review and editing (supporting). **Itsuro Koizumi:** Conceptualization (equal); resources (supporting); supervision (lead); writing – review and editing (supporting).

## CONFLICT OF INTEREST

The authors declare that they have no conflict of interest.

## Supporting information


Appendix S1

Appendix S2

## Data Availability

DNA sequences: DNA Database of Japan (DDBJ) accession numbers DRA011343 and DRA011772.
