# Supplementary material for: Dietary niche breadth influences the effects of urbanization on the gut microbiota of sympatric rodents
Source: Ecol Evol. 2022 Sep 9;12(9):e9216. doi: 10.1002/ece3.9216 (PMC9463044; doi:10.1002/ece3.9216)
Supplement: Supplementary file 2 — Appendix S2 [file ECE3-12-e9216-s002.pdf]

Supplementary Material 2

Dietary niche breadth influences the effects of urbanization on the gut microbiota of sympatric rodents

Jason L. Anders[a,b*], Alexis M. Mychajliw[c], Mohamed Abdallah Mohamed Moustafa[d,e], Wessam Mohamed Ahmed Mohamed[d], Takashi Hayakawa[f,g], Ryo Nakao[d], Itsuro Koizumi[f]

[a] Graduate School of Environmental Science, Hokkaido University, N10W5 Sapporo, Hokkaido 060-0810, Japan

[b] Center for Ecological and Evolutionary Synthesis (CEES), Department of Biosciences, University of Oslo, PO Box 1066. Blindern, 0316 Oslo, Norway

[c] Middlebury College, Departments of Biology and Environmental Studies, 276 Bicentennial Way, Middlebury, VT 05753, United States

[d] Laboratory of Parasitology, Graduate School of Infectious Diseases, Faculty of Veterinary Medicine, Hokkaido University, N18W9, Sapporo, Hokkaido 060-0818, Japan

[e] Department Animal Medicine, South Valley University, 83523, Egypt,

[f] Faculty of Environmental Earth Science, Hokkaido University, N10W5 Sapporo, Hokkaido 060-0810, Japan

[g]Japan Monkey Centre, Inuyama, Aichi 484-0081, Japan

*Corresponding author: Jason L. Anders, E-mail: j.l.anders@ibv.uio.no

Bacterial Phylum level description

Firmicutes and Bacteroidota were the two most abundant phyla in all three regions of the lower GIT of both host species comprising 80% of all microbes in *A. speciosus* and 90% in *M. rufocanus* in both the natural and urban populations. Campilobacterota was the third most abundant in these same gut regions in *A. speciosus* except in the rectum of urban animals where Proteobacteria was the third most dominant phylum. Spirochaetota was the third most abundant phylum in the cecum, colon, and rectum of *M. rufocanus* with little variation in relative abundance between natural and urban populations. Interestingly, although Firmicutes, Bacteroidota, and Proteobacteria were the three most abundant phyla in the small intestine of *A. speciosus*, Firmicutes was more abundant in the natural habitat with 71% as opposed to 58% in the urban parks, while Bacteroidota showed the opposite trend with 13% and 25% in the natural and urban habitats respectively. A similar but weaker trend was found in *M. rufocanus*; Bacteroidota made up 41% and 54% in the natural and urban sites respectively, while Firmicutes comprised 34% and 26% of all microbes in the natural and urban sites respectively.

Differential abundance analyses using ANCOM-II

A secondary differential abundance analyses was conducted comparing the relative abundance of microbial genera in each gut region between natural and urban populations using ANCOM-II as described by Kaul et al. (2017) and the R-script written by Lin (2019). We found that few microbial genera exhibited differential abundance between habitat type in either host species (Tables S12, S13). Notably, those genera with a large difference in average relative abundances such as Tyzerella (42.87%, 29.19% in natural and urban respectively) and Lactobacillus (2.44%, 8.15% averaged in natural and urban respectively) in the small intestine of *A. speciosus* were not identified as differentially abundant (Table S10). This in combination with the significantly different microbial structure between urban and natural habitats based on our beta-diversity analyses (Tables S8, S9) indicate that ANCOM-II may be too conservative an analysis for the present study.

References

Kaul A, Mandal S, Davidov O, Peddada SD. (2017) Analysis of Microbiome Data in the Presence of Excess Zeros. Front Microbiol. 8, 2114. doi: 10.3389/fmicb.2017.02114

Frederick Huang Lin (2019) Huanglin/Ancom: Third release of Ancom (v2.1). Zenodo. https://doi.org/10.5281/zenodo.3577802

Supplemental Figures

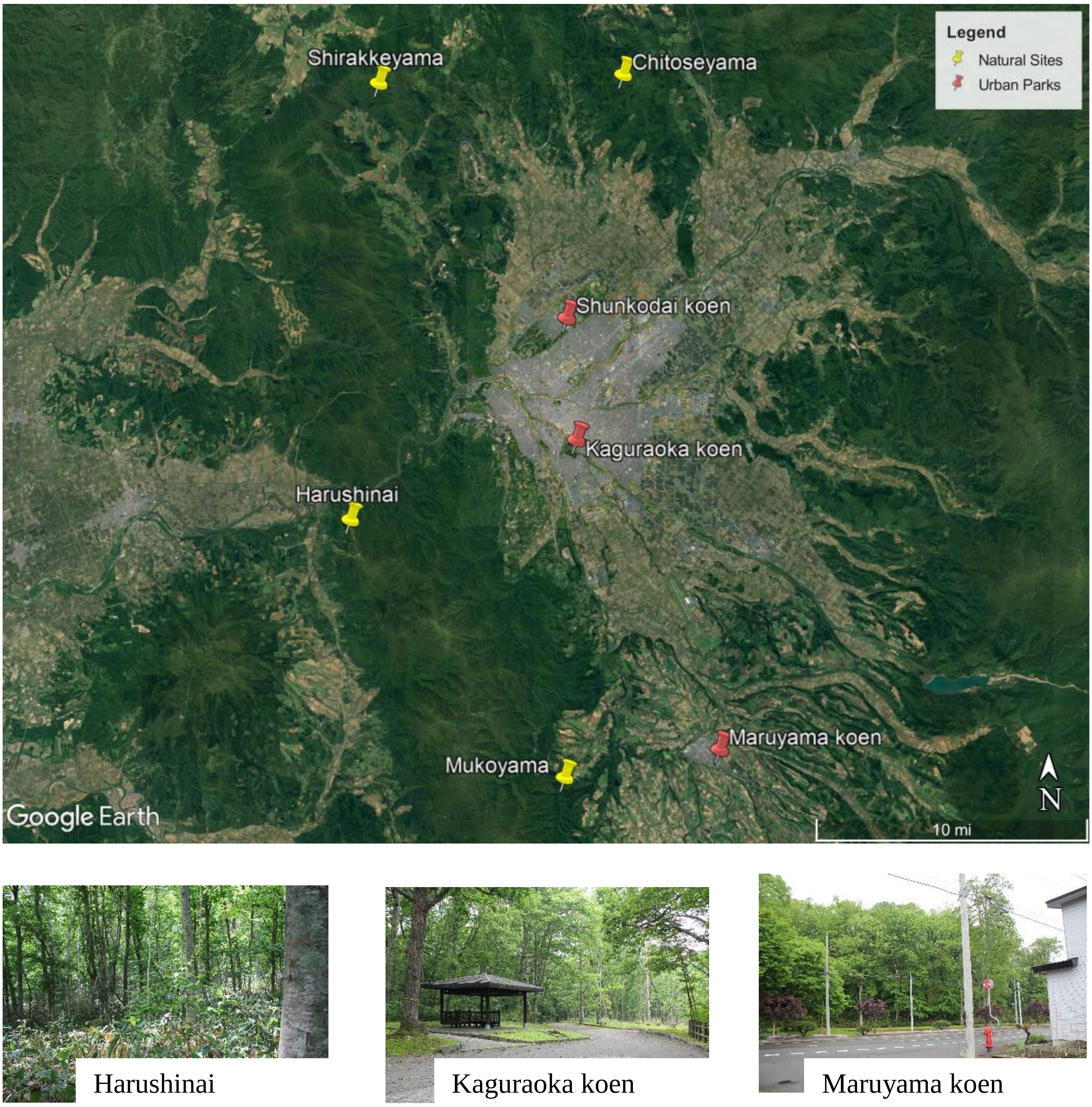

Figure S1: A satellite image showing the location of field sites and pictures of three sites (two urban and one natural). The map was created using Google Earth Pro (version 7.3.3, https://www.google.com/earth/versions/). Map data: Google, SIO, NOAA, U.S. Navy, NGA, GEBCO, © 2020 ZENRIN Image Landsat/ Copernicus

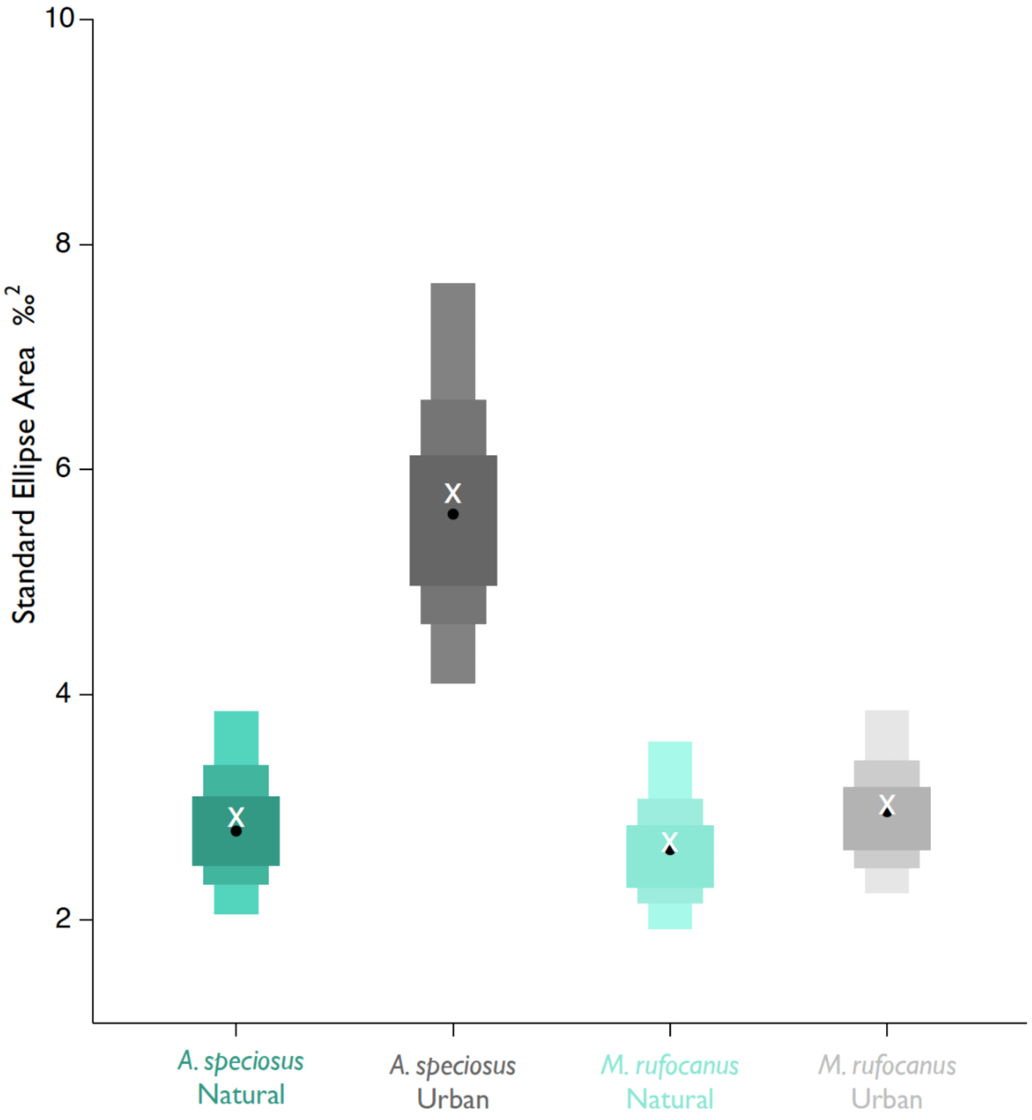

Figure S2: Bayesian standard ellipse area (SEA_b) for each group after 20,000 posterior draws where the boxes indicate 50%, 75%, and 95% confidence intervals and the white X is the maximum likelihood estimate of the small sample size corrected standard ellipse area (SEA_c).

Supplemental Tables

Habitat type	Site	Latitude	Longitude	*A. speciosus*	*M. rufocanus*
Natural	Chitoseyama	43° 56' 30.64"	142° 24' 10.09"	6	16
	Harushinai	43° 42' 19.82"	142° 12' 08.28"	5	20
	Mukoyama	43° 34' 07.41"	142° 21' 34.57"	11	3
	Shirakkeyama	43° 56' 14.15"	142° 13' 21.56"	20	4
Urban	Kaguraoka koen	43° 44' 52.24"	142° 22' 05.94"	19	13
	Shunkodai koen	43° 48' 45.38"	142° 21' 40.40"	16	29
	Maruyama koen	43° 35' 00.68"	142° 28' 23.54"	6	8
**Total**				83	93

Table S1: Site information and number of animals captured at each.

	**Small Intestine**		**Cecum**		**Colon**		**Rectum**	
**Species**	Natural	Urban	Natural	Urban	Natural	Urban	Natural	Urban
A. speciosus	42	39	27	16	41	39	25	16
M. rufocanus	43	49	14	17	38	45	16	16
**Total**	85	88	41	33	79	84	41	32

Table S2: Number of samples per gut region for both *A. speciosus* and *M. rufocanus*.

		A. speciosus		***M. rufocanus***	
		**Natural**	**Urban**	**Natural**	**Urban**
**δ^{13}C**	Mean ± StD	-27.27 ± 0.60	-27.35 ± 1.28	-25.50 ± 0.75	-24.86 ± 0.70
	Min	-28.4	-30.59	-27.36	-27.02
	Max	-26.02	-22.90	-24.08	-23.20
**δ^{15}N**	Mean ± StD	2.53 ± 1.55	3.76 ± 1.46	2.51 ± 1.17	2.22 ± 1.36
	Min	0.59	1.40	0.59	-0.04
	Max	6.22	6.43	6.56	6.60

Table S3: Summary statistics (mean, standard deviation, minimum, maximum) of stable isotopes from natural and urban populations of the two rodent species.

	Natural		Urban	
	As	***Mr***	***As***	***Mr***
**TA**	8.97	11.8	22.84	15.73
**SEA**	2.83	2.61	5.63	2.95
**SEAc**	2.9	2.68	5.77	3.01

Table S4: Estimates of isotopic niche width as measured in per mil (‰) for total area (TA), standard ellipse area (SEA), and small sample size corrected SEA (SEA_c) of natural and urban populations of *A. speciosus* (As) and *M. rufocanus* (Mr).

		A. speciosus			M. rufocanus		
**Gut region**	**Variable**	**b**	**SE**	***p***	**b**	**SE**	***p***
Small Intestine	Habitat type (Urban)	0.203	0.148	0.229	0.159	0.12	0.244
	Sex (Male)	0.181	0.139	0.198	-0.032	0.117	0.787
	Age	-0.066	0.153	0.665	-0.329	0.125	**0.01**
	$\delta^{13}C$	-0.015	0.072	0.839	-0.22	0.077	**0.006**
	$\delta^{15}N$	-0.053	0.048	0.272	0.03	0.044	0.495
Cecum	Habitat type (Urban)	0.028	0.137	0.843	-0.081	0.184	0.676
	Sex (Male)	0.194	0.066	**0.006**	0.016	0.093	0.867
	Age	-0.075	0.092	0.417	-0.179	0.095	0.075
	$\delta^{13}C$	0.03	0.032	0.363	-0.124	0.054	**0.032**
	$\delta^{15}N$	-0.029	0.024	0.229	0.043	0.034	0.224
Colon	Habitat type (Urban)	-0.001	0.055	0.988	0.06	0.056	0.335
	Sex (Male)	0.123	0.041	**0.003**	-0.054	0.039	0.163
	Age	0.079	0.046	0.088	-0.092	0.042	**0.029**
	$\delta^{13}C$	-0.022	0.026	0.399	-0.038	0.027	0.16
	$\delta^{15}N$	0.008	0.015	0.587	0.037	0.015	**0.016**
Rectum	Habitat type (Urban)	-0.028	0.091	0.771	-0.075	0.096	0.472
	Sex (Male)	0.239	0.065	**0.001**	-0.102	0.064	0.131
	Age	0.103	0.086	0.244	-0.038	0.064	0.56
	$\delta^{13}C$	0.024	0.03	0.426	-0.116	0.034	**0.003**
	$\delta^{15}N$	0.019	0.023	0.428	0.046	0.021	**0.044**

Table S5: Linear mixed effects model comparing ASV abundance diversity of the gut microbiome within each gut region between natural and urban populations. Bolded values indicate significance.

Gut region	Variable	*A. speciosus* b	SE	*p*	*M. rufocanus* b	SE	*p*
Small Intestine	Habitat type (Urban)	0.057	0.109	0.623	-0.017	0.065	0.807
	Sex (Male)	0.09	0.075	0.236	-0.01	0.056	0.863
	Age	-0.115	0.084	0.175	-0.108	0.061	0.083
	$\delta^{13}C$	-0.016	0.041	0.698	-0.046	0.038	0.222
	$\delta^{15}N$	-0.034	0.026	0.189	-0.008	0.021	0.707
Cecum	Habitat type (Urban)	-0.047	0.082	0.591	-0.063	0.084	0.484
	Sex (Male)	0.068	0.03	**0.029**	0.011	0.048	0.828
	Age	-0.072	0.041	0.091	-0.096	0.049	0.067
	$\delta^{13}C$	0.017	0.015	0.253	-0.069	0.028	**0.023**
	$\delta^{15}N$	-0.005	0.011	0.674	0.029	0.018	0.119
Colon	Habitat type (Urban)	-0.024	0.031	0.472	0.008	0.028	0.785
	Sex (Male)	0.041	0.021	0.058	-0.026	0.022	0.252
	Age	-0.005	0.024	0.844	-0.069	0.024	**0.005**
	$\delta^{13}C$	0.001	0.014	0.969	-0.033	0.015	**0.035**
	$\delta^{15}N$	0.009	0.008	0.242	0.021	0.009	**0.019**
Rectum	Habitat type (Urban)	-0.039	0.055	0.513	-0.09	0.042	0.085
	Sex (Male)	0.082	0.032	**0.016**	-0.018	0.032	0.577
	Age	0.014	0.043	0.745	-0.051	0.032	0.133
	$\delta^{13}C$	0.011	0.015	0.472	-0.047	0.017	**0.015**
	$\delta^{15}N$	0.006	0.012	0.627	0.035	0.011	**0.004**

Table S6: Linear mixed effects model comparing Faith's phylogenetic diversity of the gut microbiome within each gut region between natural and urban populations. Bolded values indicate significance.

		A. speciosus				*M. rufocanus*			
**Gut region**	**Dissimilarity index**	**SS**	**μ^2**	**F**	***p***	**SS**	**μ^2**	**F**	***p***
Small Intestine	Unweighted unifrac	0	0	0.0012	0.975	0.0147	0.0147	4.0267	**0.039**
	Weighted unifrac	0.0266	0.0266	1.6628	0.213	0.0509	0.0509	2.5048	0.109
Cecum	Unweighted unifrac	0.0066	0.0066	3.1382	0.082	0.0004	0.0004	0.3016	0.599
	Weighted unifrac	0	0	0.0013	0.974	0.0012	0.0012	0.3888	0.549
Colon	Unweighted unifrac	0	0	0.009	0.935	0.0002	0.0002	0.0944	0.756
	Weighted unifrac	0	0	0.0022	0.971	0.0066	0.0066	1.83	0.169
Rectum	Unweighted unifrac	0.0057	0.0057	2.5459	0.118	0	0	0.0243	0.88
	Weighted unifrac	0.0001	0.0001	0.0159	0.901	0.0001	0.0001	0.027	0.882

Table S7: PERMDISP results comparing the level of within habitat type dispersion of the gut microbial community. Bolded values indicate significance.

Dissimilarity index	Variable	Small Intestine				Cecum			
		**SS**	**R^2**	**F**	***p***	**SS**	**R^2**	**F**	***p***
Unweighted Unifrac	Habitat type	0.2468	0.0238	1.8441	**0.026**	0.1872	0.0483	2.1116	**0.003**
	Sex	0.1861	0.018	1.3906	0.097	0.0825	0.0212	0.9299	0.568
	Age	0.1735	0.0167	1.2967	0.14	0.0965	0.0249	1.0879	0.283
	$\delta^{13}C$	0.1161	0.0112	0.8678	0.606	0.1061	0.0273	1.1968	0.183
	$\delta^{15}N$	0.125	0.0121	0.9338	0.464	0.0806	0.0208	0.9093	0.639
Weighted Unifrac	Habitat type	0.2383	0.0299	2.303	**0.04**	0.0616	0.0243	1.0755	0.36
	Sex	0.0755	0.0095	0.73	0.624	0.0598	0.0236	1.0438	0.375
	Age	0.0932	0.0117	0.901	0.484	0.1548	0.0611	2.7049	**0.016**
	$\delta^{13}C$	0.0868	0.0109	0.8385	0.495	0.0475	0.0188	0.8305	0.565
	$\delta^{15}N$	0.0399	0.005	0.3859	0.909	0.0378	0.0149	0.6602	0.75
		**Colon**				**Rectum**			
**Dissimilarity**	**Variable**	**SS**	**R^2**	**F**	***p***	**SS**	**R^2**	**F**	***p***
Unweighted Unifrac	Habitat type	0.2481	0.0347	2.8492	**0.001**	0.186	0.0472	1.9672	**0.002**
	Sex	0.1087	0.0152	1.2479	0.111	0.1132	0.0287	1.1967	0.172
	Age	0.1683	0.0235	1.9331	**0.001**	0.1198	0.0304	1.2671	0.129
	$\delta^{13}C$	0.154	0.0215	1.7692	**0.005**	0.1012	0.0257	1.0699	0.327
	$\delta^{15}N$	0.0858	0.012	0.9858	0.482	0.0771	0.0196	0.8154	0.805
Weighted Unifrac	Habitat type	0.2153	0.038	3.1725	**0.004**	0.1175	0.0423	1.7684	0.078
	Sex	0.1349	0.0238	1.9883	**0.036**	0.1205	0.0434	1.8133	0.071
	Age	0.2767	0.0488	4.0775	**0.001**	0.109	0.0393	1.6408	0.098
	$\delta^{13}C$	0.1	0.0176	1.4741	0.141	0.0515	0.0186	0.7753	0.62
	$\delta^{15}N$	0.0716	0.0126	1.0559	0.358	0.0651	0.0235	0.9796	0.442

Table S8: PERMANOVA results comparing the gut microbiome of the different gut regions between natural and urban populations of *A. speciosus*. Bolded values indicate significance.

Dissimilarity index	Variable	Small Intestine				Cecum			
		**SS**	**R^2**	**F**	***p***	**SS**	**R^2**	**F**	***p***
Unweighted Unifrac	Habitat type	0.2815	0.0255	2.3093	**0.002**	0.1287	0.0469	1.454	**0.046**
	Sex	0.0867	0.0079	0.7113	0.905	0.0984	0.0359	1.1119	0.233
	Age	0.2114	0.0192	1.7346	**0.024**	0.1315	0.048	1.4855	**0.035**
	$\delta^{13}C$	0.3657	0.0332	3.0002	**0.001**	0.2389	0.0872	2.7	**0.001**
	$\delta^{15}N$	0.1406	0.0128	1.1532	0.203	0.1415	0.0516	1.5994	**0.013**
Weighted Unifrac	Habitat type	0.7677	0.0505	4.5194	**0.009**	0.0857	0.068	2.0661	**0.039**
	Sex	0.113	0.0074	0.6652	0.6	0.0666	0.0528	1.6051	0.113
	Age	0.2801	0.0184	1.649	0.144	0.0715	0.0568	1.725	0.074
	$\delta^{13}C$	0.4957	0.0326	2.9186	**0.03**	0.0735	0.0583	1.7714	0.086
	$\delta^{15}N$	0.1435	0.0094	0.845	0.462	0.0382	0.0303	0.9203	0.507
		**Colon**				**Rectum**			
**Dissimilarity**	**Variable**	**SS**	**R^2**	**F**	***p***	**SS**	**R^2**	**F**	***p***
Unweighted Unifrac	Habitat type	0.1915	0.0266	2.2349	**0.001**	0.1269	0.0487	1.5046	**0.037**
	Sex	0.1145	0.0159	1.3367	0.058	0.1134	0.0435	1.345	0.076
	Age	0.1435	0.0199	1.6747	**0.011**	0.118	0.0453	1.3996	0.052
	$\delta^{13}C$	0.23	0.0319	2.6839	**0.001**	0.2145	0.0824	2.5438	**0.001**
	$\delta^{15}N$	0.141	0.0196	1.6449	**0.007**	0.125	0.048	1.4815	**0.039**
Weighted Unifrac	Habitat type	0.1296	0.0344	2.7989	**0.014**	0.0892	0.0678	2.0539	0.064
	Sex	0.0424	0.0113	0.9164	0.453	0.099	0.0752	2.2793	**0.04**
	Age	0.0744	0.0198	1.6077	0.109	0.0598	0.0454	1.3761	0.203
	$\delta^{13}C$	0.035	0.0093	0.755	0.618	0.0697	0.053	1.6049	0.137
	$\delta^{15}N$	0.0363	0.0096	0.7837	0.608	0.0212	0.0161	0.4876	0.883

Table S9: PERMANOVA results comparing the gut microbiome of the different gut regions between natural and urban populations of *M. rufocanus*. Bolded values indicate significance

A. speciosus					
		**Natural**		**Urban**	
**Gut region**	**Microbial genus**	**Avg. relative abundance (%)**	**LDA**	**Avg. relative abundance (%)**	**LDA**
Small Intestine	Tyzzerella	42.87	4.83	29.19	
	Mycoplasma	10.4	4.62	1.97	
	Lactobacillus	2.44		8.15	4.53
	Mucispirillum	0.04	3.55	0.02	
	Oscillospirales UCG_010	< 0.01		0.01	3.54
	Prevotellaceae UCG_003	0.10	2.73	< 0.01	
	Bifidobacterium	0		0.01	2.63
	Pseudomonas	0.07	2.59	< 0.01	
	Ochrobactrum	0		< 0.01	2.38
	Lachnospiraceae GCA_900066575	< 0.01		0.02	2.11
	Lachnospiraceae UCG_006	< 0.01		0.03	2.11
	Providencia	< 0.01	2.07	0	
	Ruminococcus	0.01		0.03	2.07
	Desulfovibrio	0.01		0.03	2.05
	Lachnospiraceae NC2004 group	0		0.02	2
Cecum	Prevotellaceae UCG_003	2.77	4.18	0.09	
	Treponema	2.07	4.02	0.47	
	Escherichia-Shigella	1.23		2.9	3.97
	Mycoplasma	1.88	3.92	0.29	
	Prevotellaceae Ga6A1 group	1.75	3.81	0.64	
	Lachnospiraceae UCG_001	0.03		0.19	2.91
	Cerasicoccus	0.03		0.17	2.64
	Butyricicoccus	0.04		0.07	2.27

Table S10: More abundant bacterial genera in the a) small intestine, b) cecum, c) colon, d) and rectum of *A. speciosus* in the natural and urban habitats based on LEfSe analysis.

A. speciosus					
		Natural		Urban	
Gut region	Microbial genus	Avg. Relative Abundance (%)	LDA	Avg. Relative Abundance (%)	LDA
Colon	Mycoplasma	2.4	3.99	0.511	
	Prevotellaceae UCG_003	1.57	3.87	0.1	
	Prevotellaceae Ga6A1 group	1.47	3.63	0.82	
	Mucispirillum	1.56	3.62	0.89	
	Treponema	1.08	3.58	0.49	
	Bifidobacterium	0		< 0.01	3.43
	Anaerostipes	0		0.29	3.13
	Faecalibaculum	< 0.01		0.07	2.6
	Parasutterella	0.04		0.1	2.49
	Butyricicoccus	0.029		0.09	2.49
	Frisingicoccus	0		< 0.01	2.05
	Oscillospiraceae NK4A214 group	0.02		0.05	2.05
Rectum	Prevotellaceae UCG_003	2.94	4.16	0.01	
	Prevotellaceae Ga6A1 group	2.26	3.9	0.78	
	Treponema	1.55	3.81	0.22	
	Mucispirillum	1.22	3.55	0.51	
	Ruminococcus	0.75		0.68	3.48
	Anaerostipes	0		0.71	3.35
	Lachnospiraceae UCG_001	0.03		0.22	3.07
	Oscillospiraceae UCG_005	0		< 0.01	2.61
	Lachnoclostridium	0.03		0.06	2.26
	Anaerovoracaceae, Family XIII, UCG_001	0		< 0.01	2.07

Table S10 (cont.): More abundant bacterial genera in the a) small intestine, b) cecum, c) colon, d) and rectum of *A. speciosus* in the natural and urban habitats based on LEfSe analysis.

M. rufocanus					
		**Natural**		**Urban**	
**Gut region**	**Microbial genus**	**Avg. relative abundance (%)**	**LDA**	**Avg. relative abundance (%)**	**LDA**
Small Intestine	Ureaplasma	9.84	4.44	4.18	
	Helicobacter	3.5	4.2	0.18	
	Campylobacteria	0.2	4.2	< 0.01	
	Mycoplasma	4.89	4.07	4.47	
	Exiguobacterium	0.01	3.8	< 0.01	
	Coxiella	0.04	3.57	0.01	
	Mucispirillum	0.02	3.22	< 0.01	
	Escherichia-Shigella	0.42	3.19	0.24	
	Monoglobus	< 0.01		0.03	3.17
	Ehrlichia	0.08	3.14	0	
	Staphylococcus	0.01	3.12	< 0.01	
	Candidatus Lariskella	0.01	2.76	< 0.01	
	Methylobacterium-Methylorubrum	0			2.21
	Lachnoclostridium	< 0.01		0.01	2.12
	Oscillibacter	0.02	2.09	0.01	
	Oscillospiraceae UCG_005	0.01	2.02	< 0.01	
Cecum	Alistipes	2.23	3.73	1.3	
	Odoribacter	0.88	3.49	0.32	
	Colidextribacter	0.99		1.28	3.24
	Erysipelatoclostridiaceae UCG_004	0.01	3.21	< 0.01	
	Eubacterium xylanophilum group	0.31	3.13	0.08	
	Anaerovoracaceae, Family XIII, AD3011	0.02	3.02	< 0.01	
	Desulfovibrio	0.21		0.35	2.95
	Lachnospiraceae, FCS020 group	< 0.01		0.03	2.89
	Tuzzerella	0.15	2.81	0.04	
	Oscillospiraceae UCG_005	0.08	2.8	0.05	
	Lachnospiraceae, NK4B4 group	< 0.01		0.06	2.73
	Blautia	< 0.01		0.03	2.73

Table S11: More abundant bacterial genera in the a) small intestine, b) cecum, c) colon, d) and rectum of *M. rufocanus* in the natural and urban habitats based on LEfSe analysis**.**

M. rufocanus					
		Natural		Urban	
Gut region	Microbial genus	Avg. relative abundance (%)	LDA	Avg. relative abundance (%)	LDA
Colon	Lachnospiraceae, NK4A136 group	5.26		7.85	4.08
	Helicobacter	1.42		2.48	3.78
	Alistipes	2.94	3.68	2.01	
	Odoribacter	0.98	3.32	0.47	
	Gastranaerophilales	0.87	3.15	0.6	
	Eubacterium xylanophilum group	0.31	3.04	0.1	
	Allobaculum	< 0.01		0.16	2.91
	Mucispirillum	0.31	2.85	0.21	
	Erysipelatoclostridiaceae UCG_004	0.01	2.67	< 0.01	
	Intestinimonas	0.19		0.25	2.46
	Peptococcus	0.11	2.35	0.07	
	Tuzzerella	0.06	2.3	0.02	
	Lachnospiraceae, NK4B4 group	0.04		0.07	2.22
	Anaerovorax	< 0.01	2.07	0	
	Oscillospiraceae UCG_005	0.05	2.04	0.03	
Rectum	Ruminococcus	1.57	3.69	0.53	
	Eubacterium xylanophilum group	1.05	3.68	0.14	
	Peptococcus	0.18	2.68	0.09	
	Oscillospiraceae UCG_005	0.12	2.67	0.03	
	Anaerovoracaceae, Family XIII, AD3011	0.03	2.54	< 0.01	
	Lachnospiraceae, NK4B4 group	0.05		0.09	2.38
	Tuzzerella	0.05	2.32	0.02	
	Bilophila	0.02	2.2	< 0.01	

Table S11 (cont.): More abundant bacterial genera in the a) small intestine, b) cecum, c) colon, d) and rectum of *M. rufocanus* in the natural and urban habitats based on LEfSe analysis**.**

A. speciosus						
			**cut-off value**			
**Gut Regon**	**Genus**	**W**	**0.9**	**0.8**	**0.7**	**0.6**
Small Intestine	Spirochaetaceae	74	FALSE	FALSE	TRUE	TRUE
Cecum	Prevotellaceae Ga6A1 group	74	FALSE	TRUE	TRUE	TRUE
Cecum	Treponema	87	TRUE	TRUE	TRUE	TRUE
Colon	Treponema	93	TRUE	TRUE	TRUE	TRUE
Rectum	PrevotellaceaeGa6A1group	76	FALSE	FALSE	TRUE	TRUE
Rectum	Treponema	92	TRUE	TRUE	TRUE	TRUE

Table S12: Differentially abundant microbial genera between natural and urban populations of *A. speciosus* using ANCOM-II. Cutoff values indicate less conservative (0.6) to more conservative (0.9) analysis in terms of controlling for false discovery rate. TRUE indicates differential abundance and FALSE indicates no differential abundance.

M. rufocanus						
			**cut-off value**			
**Gut Regon**	**Genus**	**W**	**0.9**	**0.8**	**0.7**	**0.6**
Small Intestine	Eggerthellaceae	71	FALSE	TRUE	TRUE	TRUE
Small Intestine	Christensenellaceae	71	FALSE	TRUE	TRUE	TRUE
Small Intestine	Coxiella	80	TRUE	TRUE	TRUE	TRUE
Cecum	Lachnospiraceae FCS020 group	77	FALSE	FALSE	TRUE	TRUE
Cecum	Lachnospiraceae NK4B4 group	77	FALSE	FALSE	TRUE	TRUE
Colon	Rs-E47 termite group	96	TRUE	TRUE	TRUE	TRUE
Colon	Tuzzerella	77	FALSE	FALSE	TRUE	TRUE
Rectum	xylanophilumgroup	71	FALSE	FALSE	TRUE	TRUE

Table S13: Differentially abundant microbial genera between natural and urban populations of *M. rufocanus* using ANCOM-II. Cutoff values indicate less conservative (0.6) to more conservative (0.9) analysis in terms of controlling for false discovery rate. TRUE indicates differential abundance and FALSE indicates no differential abundance.
